# Enriched Environment Attenuates Pyroptosis to Improve Functional Recovery After Cerebral Ischemia/Reperfusion Injury

**DOI:** 10.3389/fnagi.2021.717644

**Published:** 2021-09-27

**Authors:** Jingying Liu, Jun Zheng, Yang Xu, Wenyue Cao, Jinchen Wang, Biru Wang, Linyao Zhao, Xin Zhang, Weijing Liao

**Affiliations:** ^1^Department of Rehabilitation Medicine, Zhongnan Hospital of Wuhan University, Wuhan, China; ^2^Department of Neurosurgery, Renmin Hospital of Wuhan University, Wuhan, China; ^3^Department of Anesthesiology, Zhujiang Hospital of Southern Medical University, Guangzhou, China

**Keywords:** cerebral ischemia/reperfusion, inflammasome, ischemic stroke, enriched environment, neuronal pyroptosis

## Abstract

Enriched environment (EE) is a complex containing social, cognitive, and motor stimuli. Exposure to EE can promote functional recovery after ischemia/reperfusion (I/R) injury. However, the underlying mechanisms remained unclear. Pyroptosis has recently been identified and demonstrated a significant role in ischemic stroke. The purpose of this study was to explore the effect of EE on neuronal pyroptosis after cerebral I/R injury. In the current study, middle cerebral artery occlusion/reperfusion (MCAO/R) was applied to establish the cerebral I/R injury model. Behavior tests including the modified Neurological Severity Scores (mNSS) and the Morris Water Maze (MWM) were performed. The infarct volume was evaluated by Nissl staining. To evaluate the levels of pyroptosis-related proteins, the levels of GSDMD-N and nod-like receptor protein 1/3 (NLRP1/3) inflammasome-related proteins were examined. The mRNA levels of IL-1β and IL-18 were detected by Quantitative Real-Time PCR (qPCR). The secretion levels of IL-1β and IL-18 were analyzed by ELISA. Also, the expression of p65 and p-p65 were detected. The results showed that EE treatment improved functional recovery, reduced infarct volume, attenuated neuronal pyroptosis after cerebral I/R injury. EE treatment also suppressed the activities of NLRP1/NLRP3 inflammasomes. These may be affected by inhabiting the NF-κB p65 signaling pathway. Our findings suggested that neuronal pyroptosis was probably the neuroprotective mechanism that EE treatment rescued neurological deficits after I/R injury.

## Introduction

Stroke is a disease with the highest mortality and disability rates in the world. Ischemic stroke is responsible for the majority of strokes (Campbell et al., 2019; Stinear et al., 2020). Despite the increasing improvement in the treatment of stroke, many survivors remain with residual functional deficits (Winstein et al., 2016). Therefore, the need for effective stroke rehabilitation is essential for the patients to deal with life challenges after stroke.

Enriched environment (EE) is a complex containing social, cognitive, and motor stimuli (Kempermann, 2019). EE provides laboratory animals with greater living space, further sensory stimulation, more possibilities for social interaction, and increasing opportunities for learning than the standard environment (Dahlqvist et al., 2003). The benefits of EE in neurological diseases have been extensive studied (Begenisic et al., 2015; Jungling et al., 2017; Song et al., 2017; Shin et al., 2018). In the EE, the ability of learning and memory has been significantly improved while the anxiety behavior was reduced (Benaroya–Milshtein et al., 2004). Meanwhile, exposure to EE enhanced the experience-dependent plasticity of the brain and promoted the recovery of cognitive and motor functions after ischemia/reperfusion (I/R) injury (Livingston-Thomas et al., 2016). EE improved cognitive function via neurogenesis and angiogenesis by regulating the activation of PI3K/AKT/GSK-3/β-catenin signaling pathways and intrinsic axon guidance molecules following I/R (Zhan et al., 2020). Moreover, EE mediated neurogenesis by inhibiting the production and secretion of IL-17A from astrocyte *via* NF-κB/p65 after I/R injury (Zhang et al., 2018). EE also facilitated cognitive recovery through remodeling bilateral synaptic after ischemic stroke (Wang C. et al., 2019). However, there has been little research on the relationship between EE-mediated ischemic stroke recovery and cell death. Evidence has shown that EE reduced spontaneous apoptotic cell death in the rat hippocampus (Young et al., 1999). A recent study has demonstrated that enriched environment-induced neuronal autophagy boosted the post-stroke recovery of neurological function (Deng et al., 2021). Our previous studies have illustrated that EE reduced neuronal apoptosis conducting to the superior recovery after I/R injury (Chen et al., 2017). However, the mechanisms by which EE attenuated cell death following stroke remained unclear.

Pyroptosis is a type of lytic cell death that features cell swelling, rapid rupture of the plasma membrane, and release of proinflammatory intracellular contents as a result of cleaving pore-forming proteins gasdermin D (GSDMD) following by activation of inflammasomes (Shi et al., 2017). Inflammasomes are large multimolecular complexes formed of a cytosolic sensor (nucleotide-binding domain and leucine-rich-repeat-containing [NLR] Pyrin domain containing NLRP1 and NLRP3), an adaptor protein (apoptosis-associated speck-like protein containing a CARD [ASC]), and an effector caspase pro-caspase-1 (Rathinam and Fitzgerald, 2016). After ischemic stroke attacks, the expression of inflammasomes is abundant in the brain (Abulafia et al., 2009). Pro-caspase-1 is activated through NLRP1 and NLRP3 signal cleaving GSDMD into the N-terminal gasdermin-N domain and the C-terminal gasdermin-C domain (Shi et al., 2015; Wang et al., 2020b). Then the pore-forming GSDMD-N domain causes membrane lysis inducing pyroptosis (Ding et al., 2016; Sborgi et al., 2016). Also, activated caspase-1 mediates the maturation of Interleukin-1β (IL-1β) and Interleukin-18 (IL-18) which are released into the extracellular environment subsequently (Brough and Rothwell, 2007). In recent years, increasing evidence indicated that inflammasome-mediated pyroptosis following ischemic stroke performed a crucial role in the course of functional recovery (Xu et al., 2019; Li et al., 2020). In addition, the activation of inflammasome was considered an essential step for neuroinflammation in subsequent brain injury (Walsh et al., 2014). Increasing expression of NLRP1 and NLRP3 inflammasome has been confirmed in neurons, microglia, and astrocytes (Barrington et al., 2017). Particularly, NLRP1 and NLRP3 inflammasome-mediated neuronal pyroptosis performed an increasingly crucial role in the course of ischemic stroke (Yang-Wei Fann et al., 2013; Ito et al., 2015). As a widely studied inflammation-associated transcriptional element, NF-κB regulated numerous genes and signaling pathways associated with inflammation (Afonina et al., 2017). Furthermore, emerging evidence suggested that the elevated expression of NLRP1 and NLRP3 inflammasome proteins could be modulated by the NF-κB signaling pathway in ischemic stroke (Gross et al., 2011; Fann et al., 2018). Although the accumulated evidence has shown that pyroptosis was involved in ischemic stroke injury, the relationship between neuronal pyroptosis and EE-mediated functional recovery following ischemic stroke was still unknown.

Since EE was neuroprotective and pyroptosis was involved in the progress of ischemic stroke. We formulated the hypothesis that post-stroke neurological outcomes could be improved by EE treatment to attenuate neuronal pyroptosis. In the current study, we investigated pyroptosis-related protein expression levels in the penumbra in a rat I/R injury model. Additionally, EE decreased the expression levels of NLRP1, NLRP3, and GSDMD-N. We firstly demonstrated that EE rescued neurological deficits after I/R injury involving the suppressing of neuronal pyroptosis. Generally, our findings indicated EE as a promising therapeutic method for ischemic stroke-mediated inflammasome activity.

## Materials and Methods

### Animals

**FIGURE 1:**
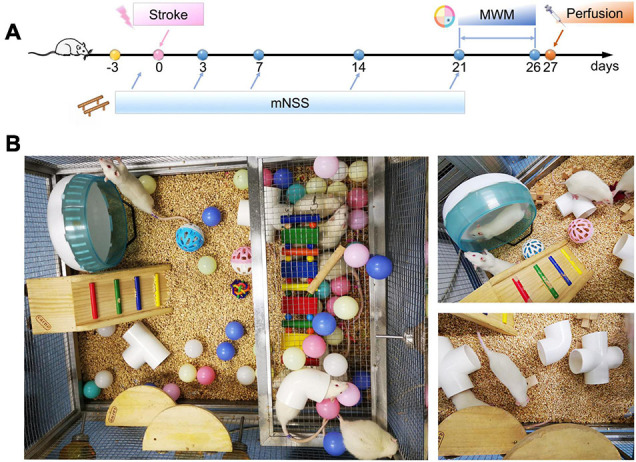
Flow chart of the experimental protocol and enrichment settings. **(A)** Timeline of behavioral testing after stroke. Modified Neurological Severity Scores (mNSS) were tested 3, 7, 14, and 21 days post-stroke (arrows) and compared with baseline performances to evaluate sensorimotor deficits. Morris Water Maze (MWM) test was performed from day 21 to day 26 post-stroke to evaluate spatial learning and memory. **(B)** The setting of an enriched environment.

Male Sprague–Dawley rats (6–7 weeks old, 200–220 g) from Beijing Vital River Laboratory Animal Technology Company were kept in individually ventilated cages (temperature: 20 ± 1°C, relative humidity: 55 ± 5%, lighting period: 8:00 ∼ 20:00) with free access to water and rat feed. Upon arrival, all rats had a 3-day acclimation before receiving the operation. Following acclimation, the animals were numbered and randomly divided into various groups: the sham + standard condition group (SSC), the sham + enriched environment group (SEE), the ischemia/reperfusion + standard condition group (ISC), and the ischemia/reperfusion + enriched environment group (IEE). The schematic representation of the experimental timeline and the setting of EE were shown in Figure 1. All animal experimental procedures were approved according to the Animal Care and Use Committee of Wuhan University. All efforts were made to minimize the mortality of animals and their suffering.

### Middle Cerebral Artery Occlusion and Reperfusion

Following adapting, male rats were subjected to transient Middle Cerebral Artery Occlusion and Reperfusion (MCAO/R) injury as previously described (Longa et al., 1989). All experimental animals were anesthetized by isoflurane through a face mask (inducing concentration: 4%, maintaining concentration: 2%, respectively, in 2:1 N_2_O:O_2_). An approximately 2 cm incision was made in the middle of the neck. The common carotid artery (CCA), internal carotid artery (ICA), and external carotid artery (ECA) were meticulously separated. And a 5–0 silk thread was used to ligate the left ECA. Then ligating the CCA with 5-0 silk thread, and clamping it at the bifurcation of the ICA with a blood vessel clip. The CCA was cut, and a monofilament nylon filament (Cinontech) was gently inserted into the ICA to approximately 18–20 mm distal to the carotid artery bifurcation. Then the left MCA was occluded. After 90 min, the filament was carefully removed to initiate reperfusion. All surgery procedures except insertion of the nylon filament were performed on rats in the sham-operation group. After recovering from anesthesia, all rats were assessed by a five-point neurological deficit score in a blinded fashion (Longa et al., 1989). Rats with scores of 1–3 points were included in this study, while the rats with scores of 0 or 4 were excluded from the study. *N* = 18/group in this study. All the experimental procedures *in vivo* were approved by The Animal Care and Use Committee of Wuhan University.

### Housing Conditions

Twenty four hour after MCAO/R, the rats were returned to their respective housing conditions. The rats of the SSC, ISC groups were kept in the standard conditions (SC) while the rats in the SEE, IEE groups were kept in the enriched environment. The details of SC and EE were as follows:

#### Standard Conditions

The rats were kept in individually ventilated cages (length: 44 cm, width: 32 cm, height: 20 cm) with bedding for animals inside. Three rats were kept in one cage.

#### Enriched Environment

The animals are placed in a stainless-steel net cage (length: 75 cm, width: 90 cm, height: 50 cm) which contained ladders, platforms, swings, colorful balls, different-shaped wooden blocks, plastic tunnels, and a running wheel for sensorimotor stimulations. And 6–10 rats were grouping housed in the EE for social stimulations. The type and location of the items in the cage were changed three times a week to ensure novelty and exploration (Figure 1B).

### Behavioral Tests

Modified Neurological Severity Scores (mNSS) (Chen et al., 2001), an 18-point scoring system compositing of motor, sensory, reflex, and balance tests, was utilized 1 day before surgery and on day 3, 7, 14, and 21 post-stroke to evaluate sensorimotor deficits (*n* = 12/group).

For spatial learning and memory testing, Morris Water Maze (MWM) test was performed on days 21–26 following I/R in a blinded situation (Morris, 1984). A round black platform (9 cm diameter, 30 cm height) was concealed in a pool (150 cm diameter, 60 cm deep, water temperature: 20 ± 1°C). On day 1–5, rats were dropped into the water from four different quadrants in turn while the position of the platform was fixed. The mean of escaping tendency to the platform in the four trials was recorded. The rat was required to stay on the platform for 15 s when reaching the platform within 60 s. The rat was guided to the platform for 15 s when reaching the platform exceeding 60 s. On day 6, the rats underwent the probe trial that allowed them to swim freely for 60 s without the platform. Swimming trajectories and the average times to reach the submerged platform were captured using an Animal Video Tracking Analysis System (*n* = 12/group) (Anilab Scientific Instruments Co., Ltd., China).

### Nissl Staining

After being fixed with 4% paraformaldehyde, tissues were embedded in paraffin cut into seriatim 4-μm-thick coronal sections with adjacent sections separated by 400 μm. The sections were placed in xylene, xylene, xylene, 100, 95, and 80% ethanol for 5 min each and rinsed under running water for 5 min. Then the sections were treated with Cresyl Violet Solution (Servicebio, China) for 3min. After washing in running water and drying thoroughly, the sections were coverslipped with neutral resin. The stained sections were scanned and measured with the ImageJ software. The total infarct volume was calculated by the formula as previously described (*n* = 6/group) (Chen et al., 2017).

### Western Blotting

Protein samples were harvested from penumbra. Tissues were ground separately in RIPA buffer comprising protease and phosphatase inhibitors (cocktails and PMSF from Aspen) for 30 min at 4°C. A BCA kit (Aspen) was used to detect the total protein concentration of each sample. Proteins were processed by SDS-PAGE (10–12.5%) and electro-blotted onto a PVDF membrane. And the membrane was then incubated in blocking buffer (5% skim milk) for 1 h at room temperature and incubated with primary antibodies including GSDMD (Abclonal), NLRP1, NLRP3, Caspase-1 (Novus), IL-1β,IL-18 (R&D), total p-65 (Proteintech),phosphorylated p-65 (Abclonal), GAPDH (Proteintech) overnight at 4°C. After washing three times, the membrane was incubated in secondary antibody for 1 h at 24°C. The proteins were scanned with a Bio-Rad system. ImageJ software was used to quantify protein levels which were normalized to GAPDH (*n* = 6/group).

### Immunofluorescence Assays

Brain paraffin sections (4 μm) were hydrated and Tris/EDTA buffer performed heat-mediated antigen retrieval for 20 min. The sections blocked with 5% BSA for 1 h were incubated with Neun (Proteintech) along with primary antibodies Caspase-1 (Novus) overnight at 4°C and subsequently incubated in fluorescent secondary antibodies (Proteintech) for 1 h at 24°C. DAPI (Antgene) was used for nuclei staining. Images were taken with an Olympus BX53 microscope (Olympus). Positive cells were counted using ImageJ software (*n* = 6/group).

### Immunohistochemistry

Brain paraffin sections (4 μm) were hydrated and Tris/EDTA buffer performed heat-mediated antigen retrieval for 20 min. The sections were then processed with 3% H_2_O_2_ for 10 min. The sections blocked with 5% BSA for 1 h were incubated with primary antibodies GSDMD (Abclonal), phosphorylated p65 (Abclonal) overnight at 4°C, and then incubated in HRP-labeled secondary antibodies (Proteintech). DAB (Servicebio) was utilized for dyeing while hematoxylin was used for nuclei staining. Images were acquired using the Olympus BX53 microscope (Olympus). The distribution and intensity of GSDMD and p-p65 staining was described by a semiquantitative score in a blinded fashion (0-negative, 1-weak, 2-moderate, 3-strong, and 4-strong and widely distributed) (*n* = 6/group) (Xu et al., 2020).

### Enzyme-Linked Immunosorbent Assay (ELISA)

Rat (*n* = 3/group) penumbra tissues were separated and homogenized with PBS. After being centrifuged at 5000 rpm for 10 min at 4°C, the supernatants were collected. The secretion levels of inflammatory cytokines (IL-1β and IL-18) were analyzed by ELISA (Elabscience). Following the instructions on the ELISA kit, the optical density (OD) at 450 nm was measured by an enzyme-labeled instrument (PerkinElmer Singapore Pte. Ltd).

### Quantitative Real-Time PCR

Rat (*n* = 3/group) penumbra tissues were separated and homogenized with Trizol reagent (Invitrogen, United States). The PrimeScript RT Reagent Kit (RR047A, Takara, Japan) was used for the reverse transcription of RNA. According to the manufacturers’ protocol, we performed qPCR to detect the mRNA levels using SYBR Premix Ex Taq II (RR820A, Takara) in a 2.1 Real-Time PCR System (Bio-Rad, United States). The relative Ct method was adopted to compare the data and GAPDH was set as internal control. The primer sequences were listed as follows:

IL-1β (F): TGACTTCACCATGGAACCCG

IL-1β (R): TCCTGGGGAAGGCATTAGGA

IL-18 (F): TGACAAAAGAAAGCCGCCTG

IL-18 (R): ATAGGGTCACAGCCAGTCCT

GAPDH (F): CGCTAACATCAAATGGGGTG

GAPDH (R): TTGCTGACAATCTTGAGGGAG

### Statistical Analysis

SPSS 23.0 software and GraphPad Prism 8.0 were used for data analysis. Analysis of mNSS was implemented by a non-parametric Kruskal–Wallis test. Analysis of escape latency in the MWM test was implemented by two-way repeated-measures ANOVA followed by Tukey’s *post hoc* test. And differences between groups were compared by two-tailed Student’s *t*-test and one-way ANOVA followed by Tukey’s *post hoc* test. All experimental data are expressed as mean ± standard deviation (SD). Statistical significance was determined as *p* < 0.05.

## Results

### Enriched Environment Improved Long-Term Neurobehavioral Function After Ischemia/Reperfusion Injury

**FIGURE 2:**
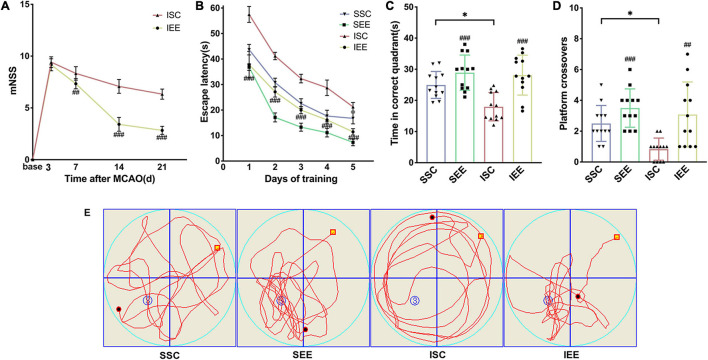
Enriched environment improved long-term neurobehavioral function of MCAO/R rats. **(A)** The mNSS of ISC and IEE group. Rats were tested before MCAO surgery. **(B)** The escape latency in the spatial learning phase. **(C,D)** Time in the correct quadrant and the crossovers in the target quadrant was recorded and analyzed. **(E)** Representative swimming trajectories of SSC, SEE, ISC, and IEE group in the probe trials. *n* = 12. Data are expressed as mean ± SD. *p < 0.05 vs. SSC group; ^##^*p* < 0.01,^ ###^*p* < 0.001 vs. ISC group.

Ischemia/reperfusion injury caused marked Behavioral dysfunction (Yirmiya and Goshen, 2011). To determine whether EE treatment improved functional recovery after I/R injury, a series of behavioral tests were performed. To evaluate the neurological function, mNSS was assessed on day 3, 7, 14, 21 after I/R injury. Rats had persistent sensorimotor defects after MCAO/R operation and EE treatment could effectively reverse the defects (Figure 2A; *p* < 0.001). To assess long-term spatial learning and memory functions, MWM was assessed on day 21–26 after I/R injury. In the spatial learning phase, the escape latency of rats in all groups decreased as the training days progressed. And MCAO/R rats spent more time reaching the platform compared with sham-operated rats on days 1–5 of training. However, rats housed in EE demonstrated the superior performance of shorter escape latency than rats housed in standard conditions following I/R injury (Figure 2B; *p* < 0.001). Probe trials proceeded 24 h after the final spatial learning trial. Rats of the IEE group spent more time in the correct quadrant and revealed more crossovers compared to rats of the ISC group (Figures 2C–E; *p* < 0.001 and *p* < 0.01). To sum up, EE treatment improved long-term neurobehavioral function after I/R injury.

### Enriched Environment Decreased Ischemic Infarction and Inhibited Pyroptosis After Ischemia/Reperfusion Injury

**FIGURE 3:**
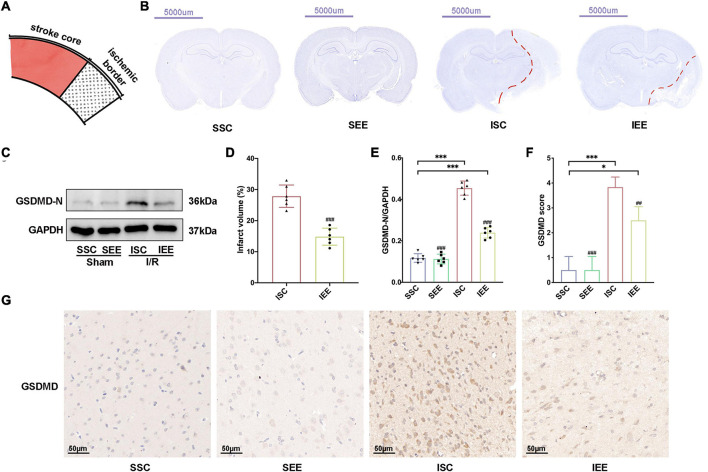
Enriched environment decreased ischemic infarction and inhibited pyroptosis after I/R injury. **(A)** Schematic brain with a highlight of the ischemic border. **(B,D)** Representative cresyl violet stained brain slices and quantification of cerebral infarct volume percentage. Scale bars, 5000 μm. *n* = 6. **(C,E)** Protein levels of GSDMD-N in peri-infarct tissues. *n* = 6. **(G,F)** Representative IHC staining images for GSDMD in the penumbra and IHC score of GSDMD in the penumbra. Scale bars, 50 μm. *n* = 6. Data are expressed as mean ± SD. *p < 0.05, ***p < 0.001 vs. SSC group; ^##^*p* < 0.01, ^###^*p* < 0.001 vs. ISC group.

As the improvement in functional outcome could be attributed to a reduction in brain damage, Nissl staining was performed to confirm the effects of EE on infarct volume after I/R injury. The schematic diagram of the ischemic border was shown in Figure 3A. EE treatment significantly reduced infarct volume in comparison with the ISC group (Figures 3B,D; *p* < 0.001). No lesion was found in SSC and SEE groups. Reduced post-stroke ischemic infarction has been reported to be associated with inhibition of pyroptosis (Ye et al., 2020). Next, we explored whether EE could rescue ischemia-induced pyroptosis. GSDMD was downstream of pyroptosis and GSDMD-N fragments transferred to the plasma membrane to form pores that caused lytic cell death and the secretion of mature IL-1β and mature IL-18 (Kovacs and Miao, 2017). The expression levels of GSDMD-N from the ischemic border were detected. Western blot results demonstrated enhanced expression levels of GSDMD-N in the ISC group. And the expression levels of GSDMD-N were apparently reduced in the IEE group in comparison with the ISC group (Figures 3C,E; *p* < 0.001).

Furthermore, we performed immunohistochemistry GSDMD and found that the IEE group expressed an obviously lower level of GSDMD in comparison with the ISC group (Figures 3F,G; *p* < 0.01).

Collectively, all of these data suggested that ischemic infarction and pyroptosis after I/R injury were negatively regulated by EE treatment.

### Enriched Environment Suppressed the Activities of NLRP1/NLRP3 Inflammasomes

**FIGURE 4:**
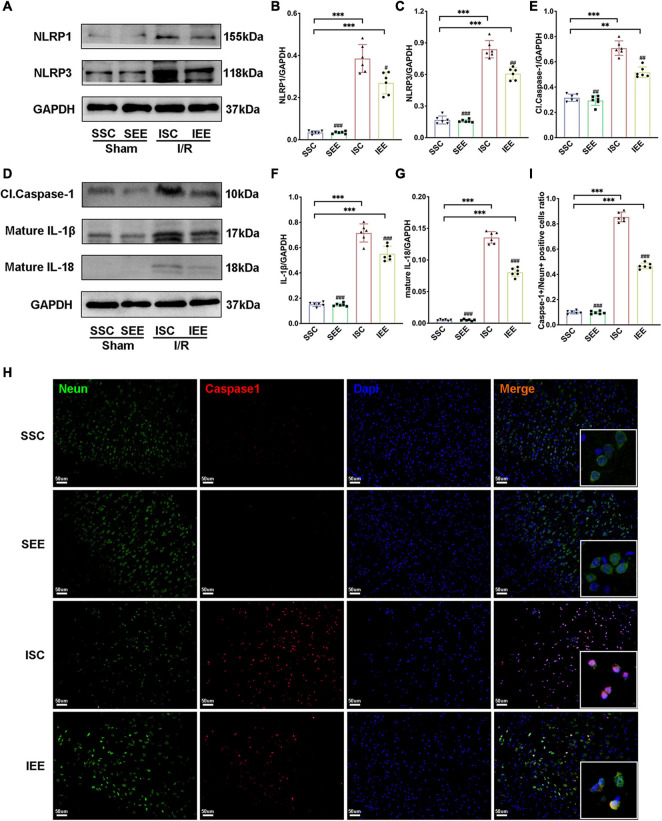
Enriched environment suppressed the activities of NLRP1/NLRP3 inflammasomes of MCAO/R rats. **(A–G)** Western blots and quantification of NLRP1/NLRP3 inflammasomes related proteins including NLRP1, NLRP3, cleaved caspase-1, mature IL-1β, and IL-18 in peri-infarct tissues. *n* = 6. **(H,I)** Double immunostaining of Neun and Caspase-1 revealed a good co-localization of these two makers. Statistical analysis of the positive rate is shown. Treatment with EE reduced Caspase-1 positive neurons in the ischemic penumbra. Scale bars, 50 μm. *n* = 6. Data are expressed as mean ± SD. **p < 0.01, ***p < 0.001 vs. SSC group; ^#^*p* < 0.05, ^##^*p* < 0.01, ^ ###^*p* < 0.001 vs. ISC group.

**FIGURE 5:**
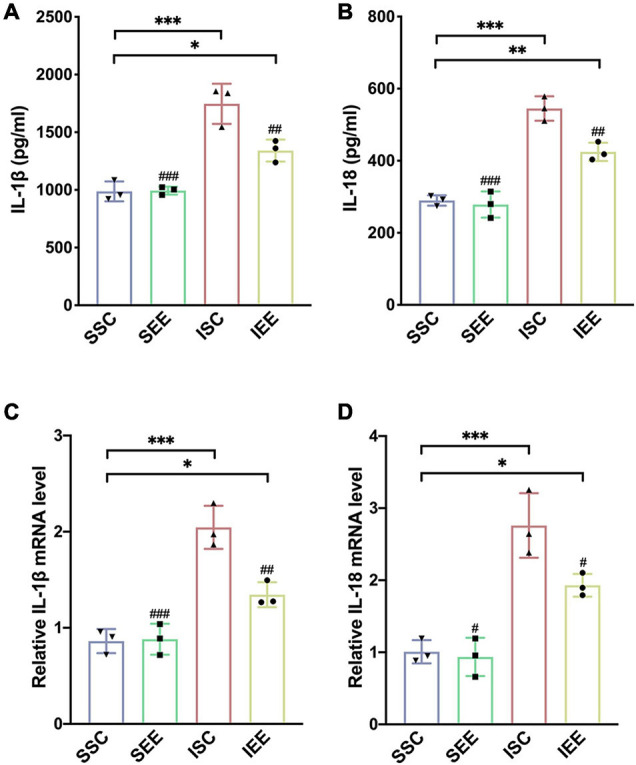
Enriched environment suppressed the expression levels of inflammatory cytokines in the penumbra. **(A,B)** Effects of EE treatment on IL-1β and IL-18 expression in penumbra according to ELISA. *n* = 3. **(C,D)** Quantitative analysis of IL-1β and IL-18 mRNA levels in penumbra. *n* = 3. Data are expressed as mean ± SD. *p < 0.05, **p < 0.01, ***p < 0.001 vs. SSC group; ^#^*p* < 0.05, ^##^*p* < 0.01,^ ###^*p* < 0.001 vs. ISC group.

To investigate how EE influenced neuronal pyroptosis after ischemic stroke, the activation of inflammasomes which was regarded as the upstream signal in the early stage of pyroptosis was detected. As neuronal pyroptosis might be mediated by the activation of NLRP1 and NLRP3 inflammasomes in the course of ischemic stroke, western blot was performed to explore whether EE inhibited pyroptosis by suppressing the activities of NLRP1/NLRP3 inflammasomes. The expression levels of NLRP1 and NLRP3 inflammasome proteins, mature IL-1β and mature IL-18 in the ischemic border of MCAO/R rats were measured. It was obvious that the expression levels of NLRP1 and NLRP3 were increased following I/R in comparison with sham controls while EE treatment decreased the expression levels of NLRP1 and NLRP3 in comparison with the ISC group (Figures 4A–C; *p* < 0.05 and *p* < 0.01). The elevated levels of cleaved caspases-1, mature IL-1β, and mature IL-18 indicated the activation of NLRP1/NLRP3 inflammasomes. I/R increased the expression levels of cleaved caspases-1, mature IL-1β, and mature IL-18 in comparison with sham controls while rats in the IEE group expressed a lower level (Figures 4D–G; *p* < 0.01, *p* < 0.001, and *p* < 0.001). To compare the expression levels of inflammatory factors, ELISAs were performed to detect inflammatory cytokines IL-1β and IL-18. We found that the expression levels of IL-1β and IL-18 were significantly increased following I/R in comparison with sham controls while EE treatment decreased the expression levels of IL-1β and IL-18 in comparison with the ISC group (Figures 5A,B; *p* < 0.01 and *p* < 0.01). The IL-1β and IL-18 mRNA expression levels were further examined by q-PCR. The mRNA levels of IL-1β and IL-18 were significantly reduced following I/R when the rats were housed in EE (Figures 5C,D; *p* < 0.01 and *p* < 0.05).

In addition, immunofluorescence analysis in the penumbra from the IEE group demonstrated a lower level of caspase-1compared with the ISC group (Figures 4H,I, *p* < 0.001). Notably, caspase-1 was highly colocalized with Neun + neurons, suggesting the inflammasome activity in neurons. These results demonstrated EE inhibited neuronal pyroptosis by suppressing the activities of NLRP1/NLRP3 inflammasomes.

### Enriched Environment Inhibited p65 Phosphorylation After Ischemia/Reperfusion Injury

**FIGURE 6:**
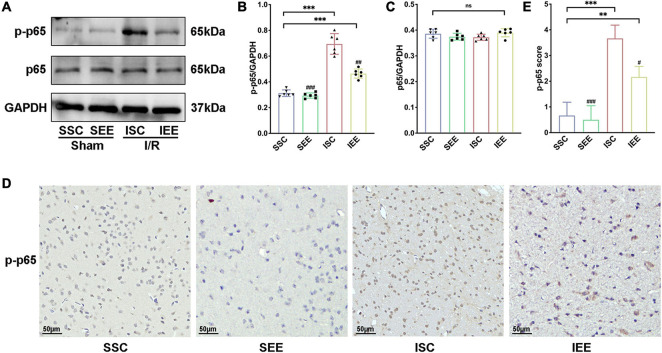
Enriched environment inhibited p65 phosphorylation after I/R injury. **(A–C)** Western blots and quantification illustrating increases in the activation of NF-κB (p-p65) in the penumbra. **(D)** Representative IHC staining images for p-p65 in the penumbra. Scale bars, 50 μm. **(E)** IHC score of p-p65 in penumbra. *n* = 6. Data are expressed as mean ± SD. **p < 0.01, ***p < 0.001 vs. SSC group; ^#^*p* < 0.05, ^##^*p* < 0.01, ^ ###^*p* < 0.001 vs. ISC group.

As NF-κB was reported to mediate pyroptosis by regulating the transcription of NLRP and then regulated its downstream substrates (Fann et al., 2018), we next explored how EE treatment inhabited pyroptosis through evaluating the NF-κB signaling pathway proteins. First, a western blot was used for the detection of p-65 and p-p65 expression levels in the penumbra following I/R injury. The results suggested that p65 levels were not influenced by EE treatment (*p* > 0.05). However, p-p65 was reduced when the rats after I/R injury were housed in EE (Figures 6A–C, *p* < 0.01). Furthermore, we used immunohistochemistry to explore the expression levels of p-p65 in the penumbra and found that rats of the IEE group significantly down-regulated p-p65 expression in comparison with rats of the ISC group (Figures 6D,E, *p* < 0.05). As is well known, p65 phosphorylation indicates the activation of the p65 NF-κB signal (Pradère et al., 2016); thus, these results illustrate that EE can inhibit the p65 NF-κB signal activation after cerebral I/R injury.

In general, these data revealed that EE treatment inhibited neuronal pyroptosis by attenuating the expression of NLRP1/NLRP3 inflammasomes following cerebral I/R injury. These may be affected by inhabiting the NF-κB p65 signaling pathway.

## Discussion

**FIGURE 7:**
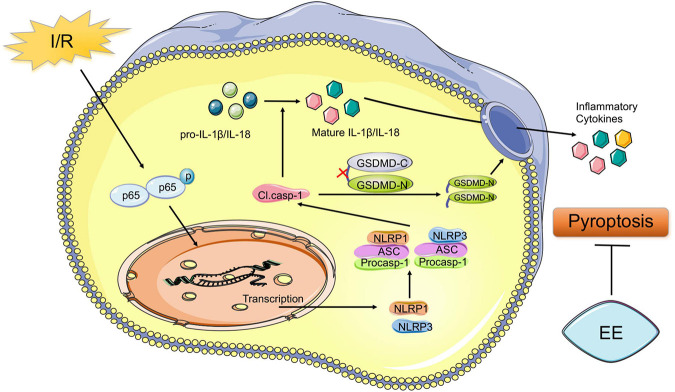
Schematic mechanism of EE treatment regulates post-ischemic pyroptosis. The NF-κB signaling pathway is activated following I/R injury, which stimulates the nucleus to induce transcription of NLRP1 and NLRP3 proteins to form the NLRP1 and NLRP3 inflammasome. Pro-caspase-1 is activated through NLRP1 and NLRP3 signal cleaving GSDMD into GSDMD-N and GSDMD-C. Then the pore-forming GSDMD-N domain causes membrane lysis inducing pyroptosis. Also, cleaved caspase-1 mediates the maturation of IL-1β and IL-18 which are released into the extracellular environment. EE attenuates pyroptosis resulting in ischemic stroke outcomes amelioration.

Despite the high rate of disability associated with stroke worldwide, valid therapeutic methods were restricted (Virani et al., 2020). Residual dysfunction of stroke greatly affected the life quality of the survivors (Stinear et al., 2020). It should not be overlooked to search methods for the recovery of the functional deficit caused by stroke. Abundant evidence showed that EE could effectively promote functional recovery after ischemic stroke (Chen J.Y. et al., 2017; Kubota et al., 2018; Lin et al., 2021, p. 1). However, due to the complexity of transforming EE into clinical practice, EE remained mainly a laboratory stage (Lang et al., 2015). Therefore, exploring the potential mechanism underlying the role of EE in promoting functional recovery may provide precise targets for the recovery of ischemic stroke and expedite its clinical application. Our previous study demonstrated the connections between neuroprotective effects of EE and neuronal cell death (Chen et al., 2017). A variety of pathological stimuli such as heart attacks, obesity, or cancer could trigger pyroptosis (Bergsbaken et al., 2009). Moreover, pyroptosis was closely related to central nervous system diseases (Fricker et al., 2018). In models of multiple sclerosis, pyroptosis inhibition preserved axons in the spinal cord lesions (McKenzie et al., 2018). In models of Alzheimer’s disease, pyroptosis relived the behavioral ability (Han et al., 2020). Previous research showed that neuronal pyroptosis affected the prognosis after ischemic stroke, which suggested that anti-pyroptosis was an effective treatment for all functional recovery following I/R injury (Lu et al., 2021). But there remained insufficient evidence that whether pyroptosis was essential for EE-mediated ischemic stroke recovery, and if so, how EE influenced neuronal pyroptosis after the pathological process. A key finding of our research was that EE attenuated pyroptosis and improved functional recovery after cerebral ischemia/reperfusion injury. The schematic mechanism was shown in Figure 7.

Being a novel type of cell death, pyroptosis mainly featured plasma-membrane pores formation, rapid plasma membrane rupture, and the release of intracellular inflammatory substances (Kuang et al., 2017). In the present study, we showed compelling evidence that the expression levels of GSDMD-N, the major pore-forming executive in pyroptosis, increased in the MCAO/R group versus the sham-operated group, and this alteration was counteracted in the EE treatment group. Then, to investigate how EE influenced neuronal pyroptosis after ischemic stroke, we detected the activation of inflammasomes which was regarded as the upstream signal in the early stage of pyroptosis. Inflammasomes were a group of the multimolecular complex that identified multiple inflammation-induced stimuli and mediate the maturation of critical proinflammatory cytokines in the process of pyroptosis (Strowig et al., 2012; Xue et al., 2019). It was worth noting that NLRP1 and NLRP3 inflammasomes were reported to be involved in ischemic stroke (Fann et al., 2014; Yang et al., 2014). However, it is not known whether EE treatment worked in the modulation of NLRP1/NLRP3 inflammasomes activation. The present research provided compelling evidence that EE treatment significantly modulated the activation of NLRP1/NLRP3 inflammasomes. The expression levels of NLRP1, NLRP3, the cleaved caspase-1, and the inflammatory cytokines mature IL-1β, and mature IL-18 were downregulated by EE treatment compared with standard conditions after I/R injury. And we found that the related proteins mainly expressed in the neurons through immunofluorescence double staining to locate its position. In summary, we found that EE treatment inhibited neuronal pyroptosis by affecting the activation of inflammasomes and thereby improved the functional recovery after I/R injury.

Next, we investigated the potential molecular mechanism of EE-reduced NLRP1 and NLRP3 inflammasome expression and activation in neurons. The activation of NLRP1 and NLRP3 inflammasome in the brain following I/R injury may be induced by pattern recognition receptors (PRRs) including toll-like receptors (TLRs), the receptor for advanced glycation end products (RAGE), and the IL-1 receptor 1 (IL-1R1) (Gelderblom et al., 2015). PRRs identified different pathological stimuli including endogenous damage-associated molecular patterns (DAMPs) released from damaged cells in the stroke core such as high mobility group box 1 protein (HMGB1), heat shock proteins, and peroxiredoxin family proteins (Tang et al., 2013; Wang et al., 2020a). DAMPs-activated PRRs further activated the intracellular NF−κB signaling pathway resulting in pyroptosis and the release of inflammatory factors (Schroder and Tschopp, 2010). As a transcription factor, NF-κB played a crucial role in cell death and inflammation (Kondylis et al., 2017; Liu et al., 2017). As a cytosolic sensor, the NF−κB signal activated and facilitated its nuclear translocation and DNA binding (Liu et al., 2017). Phosphorylation of p65 indicated the activation and functional status of the NF-κB signaling pathway (Pradère et al., 2016). Previous studies have indicated that activation of the NF-κB signaling pathway which could be activated by reactive oxygen species (ROS), hypoxia, and several inflammatory mediators occurred in neurons following I/R injury (Ridder and Schwaninger, 2009; Liu et al., 2019; He et al., 2020). The role of the NF-κB signaling pathway in regulating pyroptosis has been extensively studied in various diseases. Evidence has confirmed that NF-κB could regulate the transcription of NLRP by binding to their promoter region and then regulated its downstream substrates (Liu et al., 2017; Matias et al., 2019). The activation of the NF-κB signaling pathway was essential for the up-regulation of the protein synthesis of NLRP3 (Afonina et al., 2017). It has been demonstrated that the elevated expression level of IL-1β was induced by the activation of the NF-κB signaling pathway in ischemic damage (Zhou et al., 2012). Additionally, studies have confirmed that EE treatment was beneficial for the recovery of central nervous system diseases by inhibiting the NF-κB signaling pathway (Wu et al., 2016; Li et al., 2018). Moreover, Zhang et al. (2018) reported that EE mediated neurogenesis and functional recovery by inhibiting the NF-κB/IL-17A signaling pathway in astrocytes after ischemic stroke. In the present study, EE decreased the phosphorylation of p65 and the expression of NLRP1 and NLRP3 inflammasome proteins induced by I/R injury. This was supported by the study that NF-κB signaling promotes NLRP1 and NLRP3 inflammasome activation in neurons following I/R injury (Fann et al., 2018). In brief, our findings suggested that the anti-pyroptosis effect of EE after ischemic stroke was associated with the inhibition of the NF-κB p-65 signaling pathway and the reduced expression levels of NLRP1 and NLRP3 inflammasome-related proteins.

Less perfection was that the upstream regulator of p65 phosphorylation remained to be explored in this study. As the activation of NLRP1 and NLRP3 inflammasome may be induced by PRRs which identified DAMPs released from dying neural cells and stimulated NF-κB translocation during the I/R process, the sources of danger signals that promoted inflammatory response remained to be further investigated (Dong et al., 2018). Downregulation of the HMGB1/TLR4/NF-κB pathway was associated with inhibition of pyroptosis (Sun et al., 2020). HMGB1 activated the NLRP3 inflammasome *via* the NF-κB signaling pathway in acute glaucoma (Chi et al., 2015). The activation of the TLR4/NF-κB signaling pathway could modulate NLRP3 inflammasome activation in inflammatory bowel disease and induce GSDMD-mediated pyroptosis in tubular cells in diabetic kidney disease (Chen et al., 2019; Wang Y. et al., 2019). Moreover, SYK expression which was downregulated by the activation of miRNA-27a could stimulate the NF-κB signaling pathway and facilitate NLRP3-mediated pyroptosis (Li et al., 2021). The previous study has shown that EE could regulate the expression of HMGB1 and mediate post-stroke angiogenesis (Chen J.Y. et al., 2017). EE was also associated with growth factors (epithelial growth factor, hepatocyte growth factor) and signaling pathways (STAT3, JNK, EKR1/2, NF-κB) expressed in the gastrocnemius muscle (Le Guennec et al., 2020). However, it remained to be explored whether EE regulated the NF-κB signaling pathway by regulating the expression of these growth factors or DAMPs. It was essential to figure out the upstream signaling pathway to reveal the underlying mechanism of the EE-mediated effect on the NF-κB activation. In our future research, we would focus on solving this problem. In another hand, to confirm the exact effect of EE on the NF-κB signaling pathway, the agonist of NF-κB should be included. Our future work would dwell on this. Moreover, NF-κB pathway activation resulted in pyroptosis and the release of inflammatory factors. In turn, these inflammatory factors may act by activating the NF-κB signaling which would keep the cells in an activated cyclic state (Vallabhapurapu and Karin, 2009).

There was increasing evidence that many cell death pathways including apoptosis, autophagy and pyroptosis were simultaneously present in the ischemic core and penumbral area, which were fine-tuned and had either beneficial, deleterious or dual roles in the progression of post-stroke brain damage (Şekerdağ et al., 2018). However, the relevant research of EE on different types of cell death following ischemic stroke was extremely limited. In our previous studies, EE performed beneficial effects by inhibiting apoptosis of neurons following I/R (Chen et al., 2017). EE treatment increased the levels of anti-apoptotic protein Bcl-2 while decreased the levels of pro-apoptotic protein Bax, cytochrome *c*, caspase-3 in the penumbra after cerebral I/R injury. Caspases, the main drivers of apoptosis, were also involved in the crosstalk between apoptosis and autophagy. Activated caspases could inhibit autophagy by degrading autophagy proteins such as beclin-1, Atg5 and Atg7 (Wu et al., 2014). A recent study also demonstrated that EE promoted autophagy by increasing the expression of beclin-1 and enhancing the lysosomal activities of lysosomal-associated membrane protein 1, cathepsin B, and cathepsin D, which eventually boosted neurological function recovery following ischemic stroke (Deng et al., 2021). This kind of crosstalk in EE regulated cell death pathways after stroke could also be found in autophagy and pyroptosis. The evidence for the role of NLRP1/3 inflammasomes and pyroptosis in stroke pathology was well defined in the previous studies (Yang-Wei Fann et al., 2013; Barrington et al., 2017). Recently, autophagy has been linked to the regulation of inflammatory response. In Beclin1 +/- cells, the levels of NLRP3 and cleaved caspase1 were increased and the number of cells with inflammasome were elevated, indicating that autophagy inhibited inflammasome activation through NLRP3 degradation (Houtman et al., 2019, p. 1). Meanwhile, NLRs have been shown to increase the synthesis of autophagy-related proteins and assist in the localization of autophagy proteins (Deretic, 2012). In the present study, we found that EE inhabited neuronal pyroptosis by suppressing the activities of NLRP1/NLRP3 inflammasomes after I/R injury. The cell death pathways after I/R injury were overlapping each other in several steps of the cascades and shared common features. Keeping on exploring the effect of EE on different cell death types and how these mechanisms worked individually or correlatively might bring us a step closer to a promising approach for stroke treatment. What’s more, whether EE affected pyroptosis and the cell types experiencing pyroptosis after I/R injury remained unclear. Evidence showed that pyroptosis occurring in neurons, astrocytes, and microglia was involved in the pathological process in diseases other than ischemic stroke (Jamilloux et al., 2013; Alfonso-Loeches et al., 2014; Tan et al., 2014). In this study, we proved the effect of EE on neuronal pyroptosis after I/R injury. However, it was of great importance to notice that EE might not only influence neuronal pyroptosis but also affect pyroptosis or inflammatory reaction of additional cell types including astrocytes and microglia. Our future work would dwell on revealing the pleiotropic roles of EE-inhibited pyroptosis in other cell types.

## Conclusion

Our finding showed that EE treatment presented a prospective cerebral-protective effect against I/R injury. EE treatment promoted functional recovery after I/R injury, involving inhibition of pyroptosis by suppressing the activities of NLRP1/NLRP3 inflammasomes. The beneficial effect of EE may result from the inhibition of NF-κB p65 phosphorylation. As a result, the therapeutic application of EE after I/R injury, including several potential therapeutic targets was probably a promising strategy for stroke recovery.

## Data Availability Statement

The original contributions presented in the study are included in the article/supplementary material, further inquiries can be directed to the corresponding author/s.

## Ethics Statement

The animal study was reviewed and approved by the experimental animal Ethics Committee of Wuhan University (WP2020-08052).

## Author Contributions

JL performed the research. XZ, YX, and JL were responsible for experimental design. WC, JW, and BW contributed essential reagents and tools. LZ and JZ helped in collecting and analyzing the data. JL and JZ wrote the manuscript. XZ and WL reviewed and edited the manuscript. All the authors contributed to the article and approved the submitted version.

## Conflict of Interest

The authors declare that the research was conducted in the absence of any commercial or financial relationships that could be construed as a potential conflict of interest.

## Publisher’s Note

All claims expressed in this article are solely those of the authors and do not necessarily represent those of their affiliated organizations, or those of the publisher, the editors and the reviewers. Any product that may be evaluated in this article, or claim that may be made by its manufacturer, is not guaranteed or endorsed by the publisher.
